# Sodium current inhibition following stimulation of exchange protein directly activated by cyclic-3′,5′-adenosine monophosphate (Epac) in murine skeletal muscle

**DOI:** 10.1038/s41598-018-36386-0

**Published:** 2019-02-13

**Authors:** Hugh R. Matthews, Sapphire R. X. Tan, Jonathan A. Shoesmith, Shiraz Ahmad, Haseeb Valli, Kamalan Jeevaratnam, Christopher L.-H. Huang

**Affiliations:** 1 Physiological Laboratory, University of Cambridge, Downing Street, Cambridge, CB2 3EG United Kingdom; 2 Faculty of Health and Medical Sciences, University of Surrey, GU2 7AL Guildford, Surrey United Kingdom; 3 Department of Biochemistry, University of Cambridge, Tennis Court Road, Cambridge, CB2 1QW United Kingdom

## Abstract

We investigated effects of pharmacological triggering of *exchange protein directly activated by cyclic-3*′*,5*′*-adenosine monophosphate* (Epac) on Nav1.4 currents from intact murine (C67BL6) skeletal muscle fibres for the first time. This employed a loose patch clamp technique which examined ionic currents in response to superimposed 10-ms *V*_1_ steps to *varying* degrees of depolarisation, followed by *V*_2_ steps to a *fixed*, +100 mV depolarisation relative to resting membrane potential following 40 mV hyperpolarising prepulses of 50 ms duration. The activation and inactivation properties of the resulting Na^+^ membrane current densities revealed reduced maximum currents and steepnesses in their voltage dependences after addition of the Epac activator 8-(4-chlorophenylthio)adenosine-3′,5′-cyclic monophosphate (1 µM) to the bathing Krebs-Henseleit solutions. Contrastingly, voltages at half-maximal current and timecourses of currents obtained in response to the *V*_1_ depolarising steps were unchanged. These effects were abolished by further addition of the RyR-inhibitor dantrolene (10 µM). In contrast, challenge by dantrolene alone left both currents and their parameters intact. These effects of Epac activation in inhibiting skeletal muscle, Nav1.4, currents, complement similar effects previously reported in the homologous Nav1.5 in murine cardiomyocytes. They are discussed in terms of a hypothesis implicating Epac actions in increasing RyR-mediated SR Ca^2+^ release resulting in a Ca^2+^-mediated inhibition of Nav1.4. The latter effect may form the basis for Ca^2+^-dependent Na^+^ channel dysregulation in *SCN4A* channelopathies associated with cold- and K^+^-aggravated myotonias.

## Introduction

Intracellular cellular 3′-5′-cyclic adenosine monophosphate (cAMP) is known to activate protein kinase A (PKA)-mediated phosphorylation of a wide range of regulatory cellular Ca^2+^ signalling molecules. However, recent intense interest has concerned alternative or co-existent PKA-independent activation mechanisms particularly involving exchange proteins directly activated by cAMP (Epac) in distinct critical cell physiological processes. Yet few studies report on Epac-mediated regulation of excitable membrane, as opposed to metabolic, signalling^1,2^, in particular involving ryanodine receptor (RyR) function or its downstream effects on Na^+^ channel (Nav)-mediated signalling. Those studies available were restricted to cardiac myocytes. Thus, in intact *in situ* murine ventricular and atrial myocytes, challenge by the cAMP analog 8-(4-chlorophenylthio)-2′-O-methyladenosine 3′,5′-cyclic monophosphate (8-CPT) at concentrations specifically acting on Epac as opposed to PKA^3^ inhibited voltage-dependent Na^+^ currents under loose patch clamp recording conditions under which their intracellular Ca^2+^ homeostasis conditions were thereby preserved^4^. This accompanied pro-arrhythmic reductions in action potential conduction velocities in intact perfused hearts^5^. Both actions were reversed by the ryanodine receptor (RyR) blocker dantrolene which by itself contrastingly did not alter Na^+^ currents^4^.

These findings were consistent with an action of Epac activation upon Nav1.5 through an increased RyR-mediated sarcoplasmic reticular (SR) Ca^2+^ release that would in turn modify Nav1.5 function. In murine cardiomyocytes, Epac is thought to cause a downstream RyR phosphorylation stimulating SR Ca^2+^ release thereby modifying Ca^2+^ homeostasis. Thus, the Epac activating agent, 8-CPT, elicits occurrences of spontaneous cytosolic Ca^2+^ ([Ca^2+^]_i_) transients. It also increases the amplitudes of evoked [Ca^2+^]_i_ transients following action potential excitation. Finally, it results in an appearance of spontaneous propagated cytosolic Ca^2+^ waves in rat and mouse cardiomyocytes^6^. These findings were accompanied by pro-arrhythmic extrasystolic electrophysiological events in intact perfused hearts^7–10^. Both effects persisted in the presence of the PKA inhibitor H-89^11^. However, they were abolished by genetic ablation of Epac2, β_1_-adrenoreceptors or Ca^2+^/calmodulin-dependent protein kinase II (CaMKII)-δ, as well as by RyR2-S2814 phosphorylation^12^. The resulting altered [Ca^2+^]_i_ in turn could then potentially modulate voltage-gated Na^+^ channels (Nav) that generate propagated action potentials. The intracellular C-terminus domains of cardiac Nav1.5 possess EF hand-like motifs to which Ca^2+^ can bind directly. Nav1.5 also possesses an IQ-like domain to which Ca^2+^ can bind indirectly via calmodulin (CaM) as well as phosphorylation sites for CaMKII^13^. Different reports have variously implicated all three of these sites in the modified or inhibited Nav1.5 function^14–17^ observed when intracellular Ca^2+^ was varied in patch-clamped cardiomyocytes^18^.

Skeletal myocytes represent a cell type distinct from cardiac myocytes. They express differing skeletal muscle RyR1, rather than cardiac RyR2, isoforms. These are activated by direct charge coupling as opposed to Ca^2+^-induced Ca^2+^ release, by differing surface membrane Cav1.1 as opposed to Cav1.2 L-type Ca^2+^ channel isoforms, not involving activation of membrane Ca^2+^ current^19^. These events are initiated by depolarisation driven by Nav1.4 rather than Nav1.5 channel opening. Furthermore, abnormal skeletal muscle Nav1.4 and cardiac muscle Nav1.5 function cause distinct clinical consequences. Genetic abnormalities affecting Nav1.5 potentially cause clinical cardiac pro-arrhythmic effects. Nav1.4 dysfunction is contrastingly implicated in hyperkalaemic and hypokalaemic periodic paralysis^20–22^, paramyotonia congenita^21^, cold- and K^+^-aggravated myotonia^23^, and sudden infant death syndrome^24^. Cold- and K^+^-aggravated myotonias particularly are associated with compromised Ca^2+^-mediated regulation of Nav1.4^25^.

However, in common with cardiomyocytes, skeletal myocytes possess G-protein coupled β-adrenergic receptors which generate cAMP_i_ on activation^26^. Furthermore, homologies between Nav1.4 and Nav1.5 are compatible with similarities in functional properties^13^. In preliminary reports, Nav1.4 function was inhibited by Ca^2+^ entry through neighbouring Ca^2+^ channels, photorelease of caged Ca^2+^ in transfected HEK293 cells and skeletal muscle cell lines^25^, and following release of mitochondrial Ca^2+^ in murine skeletal muscle fibres^27^. CaM overexpression similarly negatively shifted steady-state voltage-dependences of Nav1.4 inactivation. This was rescued by expressing mutant CaM with impaired Ca^2+^ binding^28,29^. However, this evidence largely derives from cultured or heterologous cell lines studied by whole-cell patch-clamp methods that themselves perturb intracellular Ca^2+^ homeostasis. Furthermore, other reports demonstrated inhibitory effects of Ca^2+^ and CaM on Nav1.4 even under conditions when they did not inhibit cardiac Nav1.5^28^. Finally, previous explorations of Epac actions in skeletal myocytes concerned actions of Epac1 in inhibiting proteolysis, inducing mitochondrial biogenesis^30^ and regulating AMP-activated protein kinase^31^. They did not study electrophysiological effects.

The present experiments explore downstream effects of Epac activation on skeletal Nav1.4 function, characterised by Na^+^ current activation and inactivation properties, through its action on RyR1-mediated release of intracellularly stored Ca^2+^ for the first time. They studied mammalian, murine, muscle, thought to share many genetic and physiological properties with *in situ* human skeletal muscle^32^. Transcriptome analyses has demonstrated close similarities between murine soleus and human skeletal muscle^33^. Mouse models are amenable to further detailed studies of recently available genetically modified murine Epac1^−/−^ and Epac2^−/−^ variants^34^. The present experiments employed the Epac activator 8-CPT and controls using the RyR inhibitor dantrolene comparable with manoeuvres previously used to explore parallel regulatory features of Nav1.5. They similarly used loose patch clamp recordings and its related pulse protocols. Such experimental configurations similarly minimised intracellular perturbations particularly those involving [Ca^2+^]_i_ homeostasis associated with conventional patch clamp methods^4^.

## Results

### Currents obtained in the combined pulse protocol

**Figure 1:**
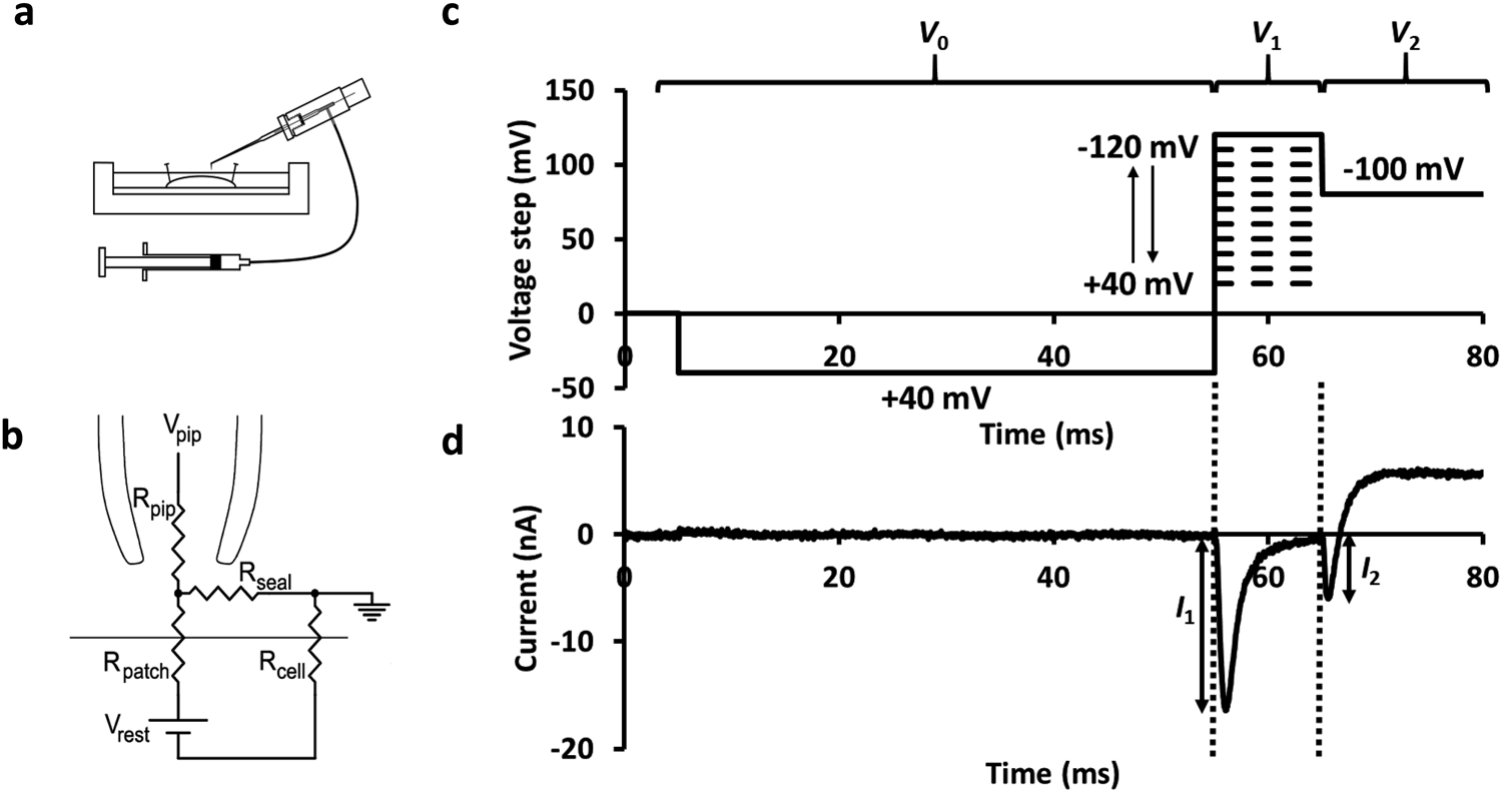
Experimental loose patch configuration: (**a**) Pinned muscle preparation under Krebs-Henseleit solution with loose patch pipette mounted at 45 degrees to the preparation but bent to permit right-angled contact of the pipette tip with the myocyte surface. Pipette connected to suction syringe. (**b**) Equivalent circuit of loose patch clamp electrode on membrane. Pipette clamped at voltage V_pip_. Compensation for the voltage error arising from currents flowing through the series combination of the pipette resistance (R_pip_) and the seal resistance (R_seal_) was achieved using a bridge circuit in the custom-designed loose patch clamp amplifier. As the loose patch technique alters the extracellular potential within the patch relative to resting membrane potential (RMP), negative *V*_0_ and positive *V*_1_ and *V*_2_ voltage excursions in V_pip_ respectively produce hyperpolarising and depolarising voltage steps relative to RMP as indicated on the ordinate. (**c**) Pulse protocol imposing three voltage steps over a time course of 80 ms before restoring RMP.  A 50 ms duration, −40 mV, hyperpolarising pre-pulse voltage step *V*_0_ maximises and standardises the initial proportion of Nav channels in the resting as opposed to inactivated state. The subsequent *V*_1_ depolarising voltage step of varying amplitudes to membrane potentials between RMP − 40 and RMP + 120 mV was used to analyse the voltage dependence of Na^+^ current activation. The final *V*_2_ voltage step to a fixed potential RMP + 100 mV permitted assessment of Na^+^ channel inactivation reflected in the presence of Na^+^ current persistent following the preceding *V*_1_ step. (**d**) Typical current trace recorded during a single sweep in the experimental protocol to determine quantitative measures of activation and inactivation. The peak currents *I*_1_ and *I*_2_ obtained during the respective voltage steps *V*_1_ and *V*_2_ measured relative to the current immediately before the corresponding voltage step provided measures of Na^+^ current activation and Na^+^ current inactivation respectively corresponding to the membrane potential at the *V*_1_ voltage step. Data from patch 2102st05.dat (sweep 18), *V*_0_ = −40, *V*_1_ = +45, *V*_2_ = +100 mV relative to the resting potential respectively.

The loose patch clamp technique alters transmembrane voltage by changing the voltage on the extracellular rather than the intracellular side of the intact membrane under study (Fig. 1a,b). Accordingly, positive voltage changes in the intrapipette potential result in membrane hyperpolarisation relative to the resting membrane potential (RMP) and negative changes in the intrapipette potential result in membrane depolarisation relative to the RMP. For clarity the signs of the voltage steps applied to the pipette have been inverted when describing the pulse protocols so that they represent the changes in the conventionally-expressed membrane potential relative to the RMP. Accordingly, in Fig. 1c, depolarising voltage steps relative to the RMP correspond to negative changes, and hyperpolarising voltage steps to positive changes in pipette potential. The patched membrane was initially held at the RMP for 5 ms following the onset of recording. A pre-pulse hyperpolarising *V*_0_ voltage step from the RMP to a membrane potential of (RMP-40) mV of duration 50 ms was applied to remove any residual Na^+^ channel inactivation that might exist at the RMP. It would maximise and standardise the proportion of Na^+^ channels in the resting state relative to the inactivated state prior to imposition of the remaining *V*_1_ and *V*_2_ voltage steps. Use of the double pulse protocol allowed an analysis of the voltage-dependences of both Na^+^ current activation and inactivation within the same protocol (Fig. 1c). Thus, depolarising *V*_1_ voltage steps of 10 ms duration were then made to membrane potentials between (RMP-40) mV to (RMP + 120) mV through successive sweeps in 5 mV increments. The currents obtained in response to these were used to investigate the voltage-dependence of Na^+^ current activation. In each sweep, this was then followed by superimposing a further *V*_2_ voltage step to a constant strongly depolarised membrane potential of (RMP + 100) mV. This would activate the remaining Na^+^ channels which had not entered the inactivated state by the end of the prior depolarising *V*_1_ step. Currents from this second step accordingly allowed characterisation of the degree of Na^+^ current inactivation induced by the preceding varying *V*_1_ steps, and thus the voltage-dependence of Na^+^ current inactivation.

Figure 1d illustrates a typical pipette current trace recorded during a single sweep in the protocol following correction for residual leakage by a P/4 protocol in which the *V*_1_ step was made to a membrane potential of (RMP + 40) mV. Inward currents are represented as downward, negative, deflections and outward currents as upward, positive, deflections. Records typically began with a small upward deflection in response to the *V*_0_ pre-pulse to a membrane potential of (RMP-40) mV. The initial *V*_1_ test voltage steps yielded early inward current transients, *I*_1_, whose amplitudes enabled quantification of activation. The subsequent *V*_2_ voltage step similarly elicited inward currents, *I*_2_, whose amplitude would be expected to vary with the inactivation expected to take place with the preceding pulse *V*_1_. The amplitudes of *I*_1_ and *I*_2_ were measured in relationship to the current recorded just before the *V*_1_ and *V*_2_ voltage steps respectively. The magnitude of *I*_1_ would represent the degree of Na^+^ current activation in response to *V*_1_ while changes in *I*_2_ would provide measures of the degree of Na^+^ current inactivation produced by the preceding *V*_1_ voltage step. The Na^+^ current amplitudes, *I*_1_ and *I*_2_, in response to the respective *V*_1_ and *V*_2_ voltage steps thus permitted quantification of the voltage-dependences of Na^+^ current activation and inactivation respectively.

**Figure 2:**
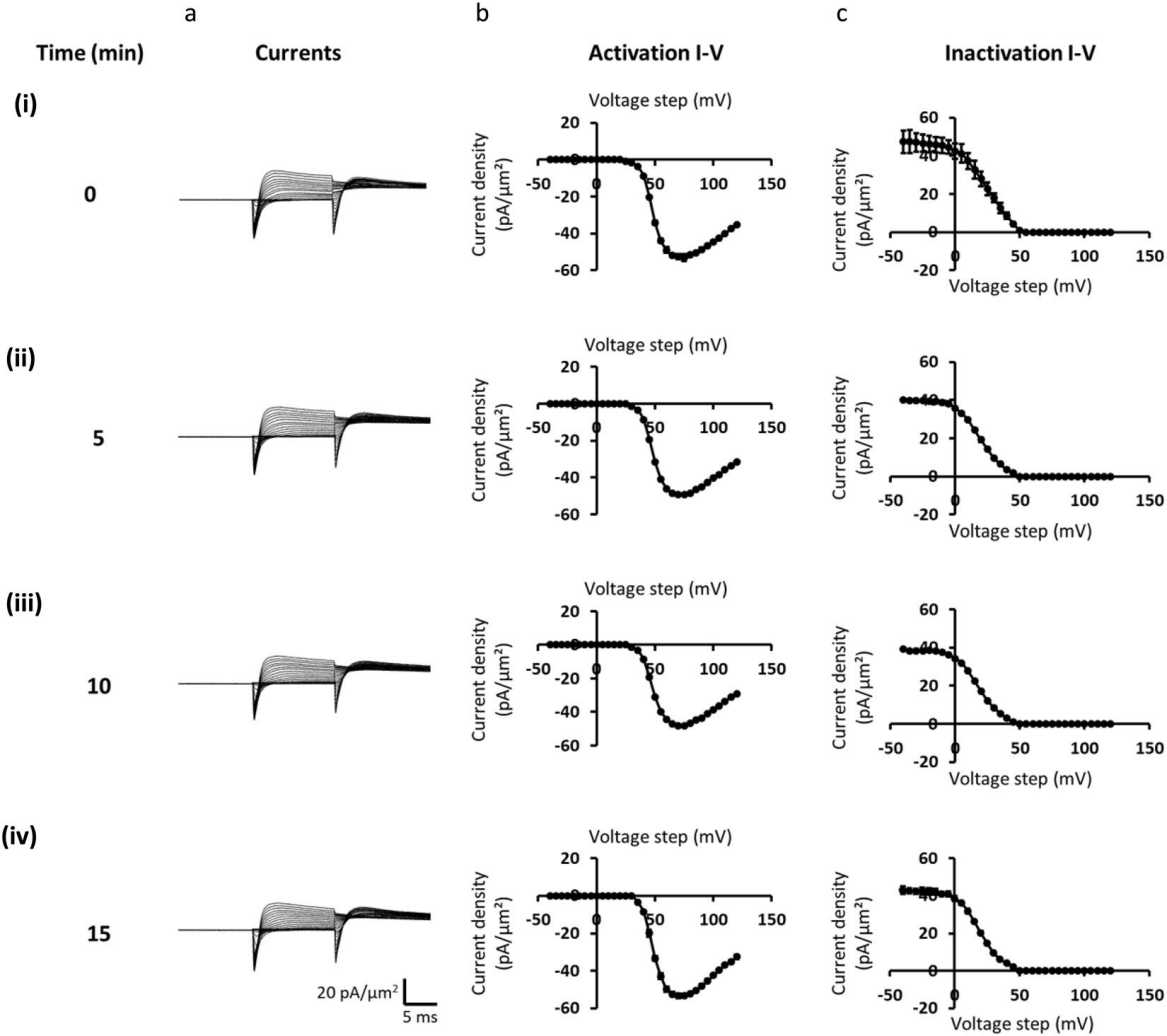
Sets of ionic currents, and activation and inactivation current-voltage relationships obtained from sequentially repeated pulse protocols through investigation of the properties of a single patch. Displays of (**a**) currents, (**b**) activation and (**c**) inactivation current voltage curves through different stages ((i)-(iv)) of the study of the single patch illustrated.

The protocols were organised to monitor and correct for any monotonic drifts in current. We thus first applied a sequence of sweeps ordered from the smallest to the largest *V*_1_ voltage steps. We then repeated this procedure in the reverse order from the largest to the smallest *V*_1_ voltage steps. The mean of the two measurements obtained at each *V*_1_ step was then calculated and used for analysis. This process was repeated four times in the examination of each patch, with the resulting traces averaged to make up experimental records for display and analysis. Figure 2 illustrates records of observed currents (Fig. 2a), and the resulting activation current-voltage (Fig. 2b) and inactivation curves (Fig. 2c) obtained from the observed peak currents *I*_1_ and *I*_2_ shown separately for each repeat. These demonstrate the consistency of the findings obtained from a patch that was studied that gave stable results throughout the duration (Fig. 2(i–iv)) of the experimental protocols.

### Comparisons of Na^+^ currents obtained before and following 8-CPT challenge

**Figure 3:**
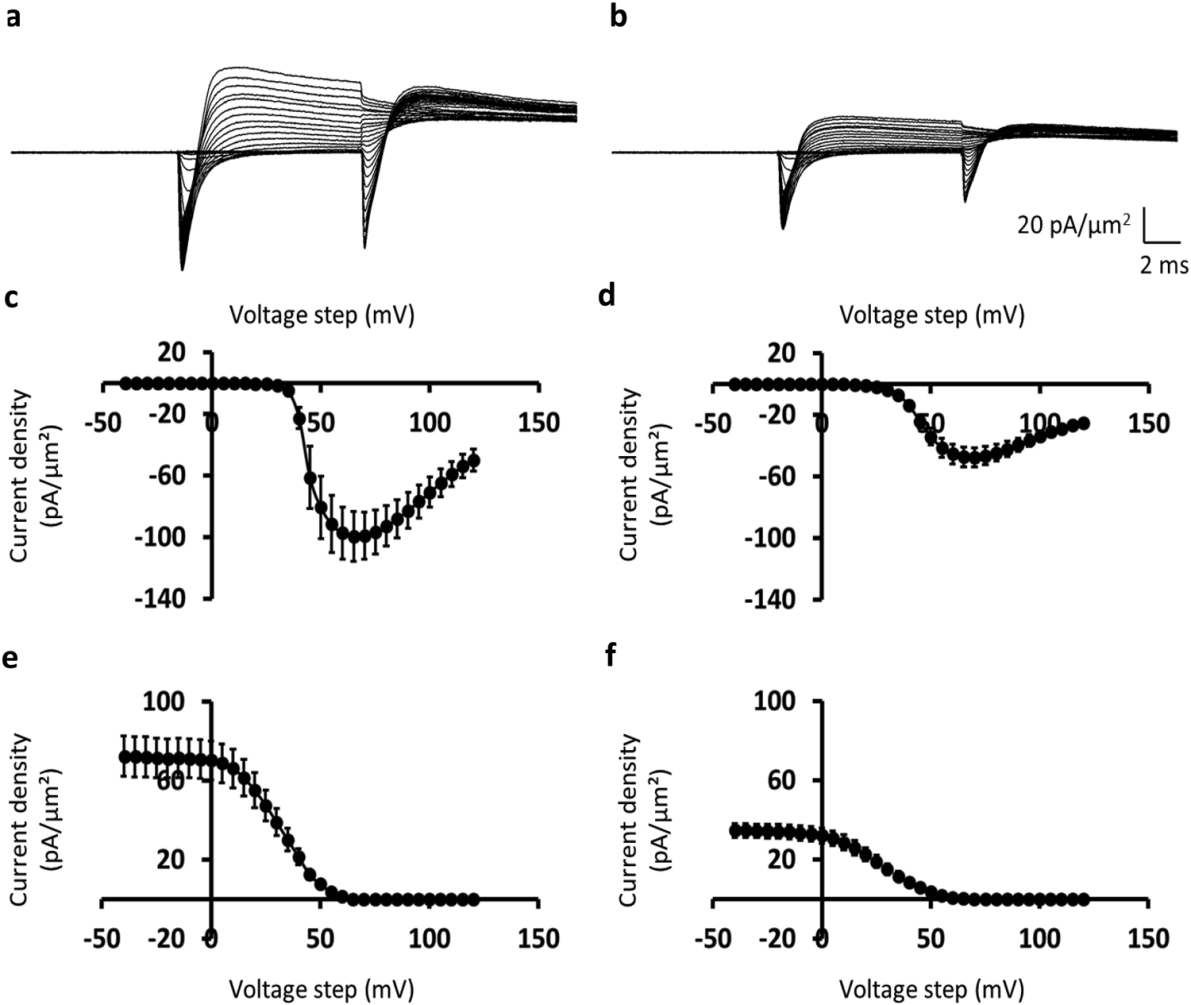
Loose-patch clamp current records obtained before and following 8-CPT challenge. (**a**,**b**) Typical families of membrane ion currents, dimensioned in pA/*μ*m^2^, observed (**a**) before and (**b**) following 8-CPT challenge in response to *V*_1_ steps to varying and *V*_2_ steps to a fixed membrane potential. Note increase in *I*_1_ and decrease in *I*_2_ with increasingly depolarising *V*_1_ steps, and reductions in both *I*_1_ and *I*_2_ with 8-CPT treatment. (**c**–**f**) Plots (means ± SEM) of peak inward currents, *I*_1_ and *I*_2_, in response to (**c**,**d**) *V*_1_ steps and (**e**,**f**) *V*_2_ steps respectively reflecting voltage dependences of inward current (**c**,**d)** activation and (**e**,**f)** inactivation (**c**,**e**) before and (**d**,**f**) following 8-CPT challenge. Data from (**a**,**c**,**e**) patch 2702st03.dat (sweeps 1–66), (**b**,**d**,**f**) patch 2702st06 (sweeps 1–66); *V*_0_ = −40, *V*_1_ = from −40 to +120, *V*_2_ = +100 mV relative to the resting potential for both data sets.

Currents were first measured before and following treatment with the Epac activator 8-CPT at a final concentration of 1 *μ*mol/L, at which it is highly specific for Epac over PKA^35^ using the same pipette and muscle preparation. Figure 3(a,b) illustrate typical loose patch clamp currents obtained from representative patches before (panel a) and following (panel b) pharmacological challenge. Currents are normalised to the area of the pipette tip lumen (*µ*m^2^) to give current densities (pA/*µ*m^2^). The pretreatment inward Na^+^ currents showed their typical activation and inactivation time courses (Fig. 3a). The initial test depolarising *V*_1_ steps elicited transient inward currents that initially increased with time to a peak value that increased non-linearly with progressive depolarisation reflecting the voltage-dependence of Na^+^ current. They then decayed reflecting channel inactivation whose extent and kinetics was similarly determined by the voltage *V*_1_. Subsequent superimposition of the *V*_2_ test steps upon the varying *V*_1_ voltage excursions to a constant strongly depolarised level (RMP + 100) mV similarly elicited inward Na^+^ currents with typical activation and inactivation time courses. However, the resulting deflections decreased in amplitude the more depolarised the *V*_1_ excursion. This is as expected for Na^+^ channel inactivation that is dependent upon, and becomes more marked with, the preceding voltage level *V*_1_. In a significant number of records the initial inward currents following *V*_1_ and *V*_2_ steps were followed by a more gradual development of an outward current expected from a delayed K^+^ current activation. However, challenge by 8-CPT markedly decreased the inward Na^+^ current amplitudes whether with the *V*_1_ or *V*_2_ voltage steps at all the test voltages explored by the *V*_1_ steps (Fig. 3b).

### Activation Na^+^ current-voltage curves before and following 8-CPT challenge

The reduction in Na^+^ currents could be represented in the plots of voltage-dependence of Na^+^ current activation following the *V*_1_ voltage step (Fig. 3c) before and (Fig. 3d) during treatment with 8-CPT. The voltage-dependence of Na^+^ current activation following the *V*_1_ voltage step during the different pharmacological conditions was then plotted in Fig. 3c,d. This was obtained from the average value of *I*_1_ determined for each patch studied and plotting the resulting Na^+^ current activation (mean of *I*_1_ ± standard error of the mean (SEM)) for each value of voltage excursion in the *V*_1_ step. The rising phases of the current-voltage (*I*-*V*) relationships from each patch obtained before and following pharmacological intervention were then characterised by fits to a Boltzmann function: *I*_1_ = *I* = *I*_max_*/*[1 + *exp*{(V* − *V*)/*k*}]), relating the peak current *I* at any given voltage excursion *V*_1_ through a maximum value of such peak current (*I*_max_)_,_ the voltage V* at half-maximal current activation, and a steepness factor term *k*. The collected *n* values were then expressed as means ± SEM and values of the parameters compared (a) before and (b) during drug treatment by unpaired 2-tailed *t*-testing to a *P* < 0.05 significance level.

The values of *I*_max,_ V* and *k* for the activation I-V curves under each pharmacological condition were expressed as means ± SEM (*n*-value). 8-CPT reduced Na^+^ current with small changes in its voltage-dependence. Thus, although there were similar voltages at half-maximal current, V* (45.61 ± 1.66 mV (*n* = 6) vs. 45.09 ± 3.35 mV (*n* = 6); *t*-statistic (*t*) = 0.13, degrees of freedom (φ) = 10, *P* ≫ 0.05), there were increased values of *k*, reflecting decreased voltage sensitivity of current activation, following addition of 8-CPT (from 3.56 ± 0.62 mV (*n* = 6) to 5.75 ± 0.57 mV (*n* = 6); *t* = 2.36, φ = 10, *P* < 0.05), and marked reductions in the values of the maximum current *I*_max_ with 8-CPT challenge (from −99.06 ± 15.19 pA/*μ*m^2^ (*n* = 6) to −38.04 ± 4.48 pA/*μ*m^2^ (*n* = 6); *t* = 3.52, φ = 10, *P* < 0.01).

### Inactivation Na^+^ current-voltage curves before and following 8-CPT challenge

Concordant observations were obtained from analysis of Na^+^ inactivation. Figure 3e,f plot the corresponding voltage-dependence of Na^+^ current inactivation produced by varying *V*_1_ voltage steps prior to the *V*_2_ test step to a fixed membrane potential (e) before and (f) following treatment with 8-CPT. Peak currents, *I*_2_, in response to the *V*_2_ step are plotted (mean of *I*_2_ ± SEM) for each value of voltage excursion in the preceding *V*_1_ step. These fell with increasing depolarisation produced by the *V*_1_ voltage step, reflecting the voltage-dependence of Na^+^ current inactivation. The dependences of *I*_2_ upon *V*_1_ for each patch were then characterised by fits to a Boltzmann function: *I*_2_ = *I* = *I*_max_(1 *−* 1*/*[1 + *exp*{(V* *−* *V*)/*k*}]), where *I* is the peak current associated with given voltage excursion *V*_1_, *I*_max_ the maximum peak current, V* the voltage at half-maximal current inactivation, and *k* the steepness factor term. The collected *n* values were then again expressed as means ± SEM and values of the parameters compared during treatment and before drug treatment by unpaired 2-tailed *t*-testing to a *P* < 0.05 significance level. The values for *I*_max_, V* and *k*, were expressed as means ± SEM (*n*-value).

Analysis of inactivation data demonstrated again that introduction of 8-CPT resulted in marked reductions in the inward current. There were similar voltages at half-maximal current, V* (30.70 ± 1.23 mV (*n* = 6) vs. 25.78 ± 2.35 mV (*n* = 9); *t* = 1.78, φ = 13, *P* ≫ 0.05), but increases in *k* (from 8.82 ± 0.20 mV (*n* = 6) to 10.24 ± 0.28 mV (*n* = 9); *t* = 4.13, φ = 13, *P* < 0.01), demonstrating slightly decreased voltage sensitivities of peak current inactivation, following addition of 8-CPT. There were also pronounced reductions in the values of the maximum current *I*_max_ with 8-CPT challenge (from 71.86 ± 9.96 pA/*μ*m^2^ (*n* = 6) to 34.61 ± 3.50 pA/*μ*m^2^ (*n* = 9); *t* = 4.48, φ = 13, *P* < 0.001).

### Na^+^ current timecourses before and following 8-CPT challenge

**Figure 4:**
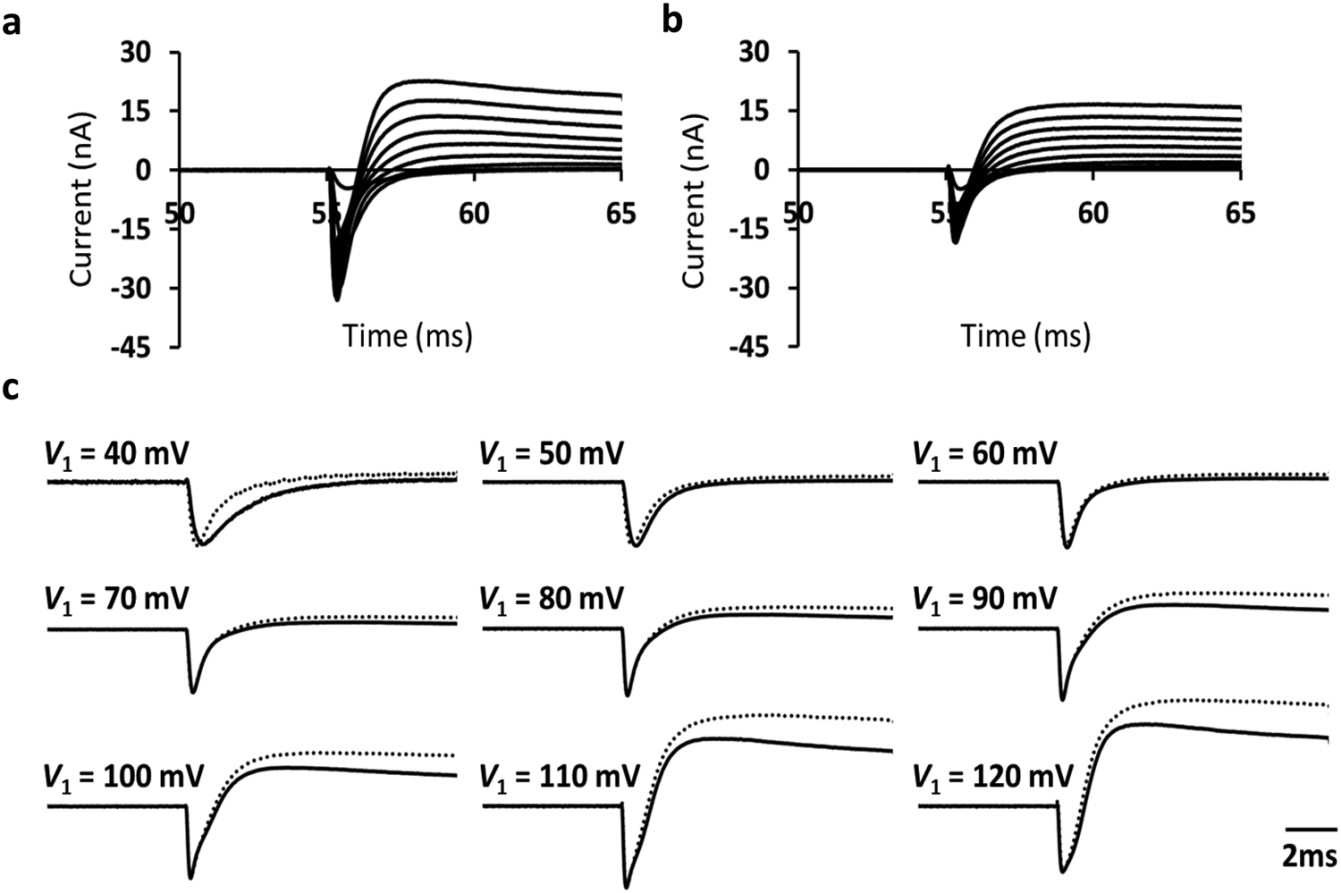
Time courses of currents before and following 8-CPT challenge. Comparison of time course of inward currents before and following 8-CPT challenge. (**a**,**b**) currents dimensioned in nA, obtained by signal averaging currents from sweeps imposing *V*_1_ steps between RMP + 40 and RMP + 120 mV in a single patch employing the pulse protocol (**a**) before and (**b**) following challenge by 8-CPT. (**c**) Superimposed traces obtained before (solid lines) and following (dotted lines) both normalised to their peak values at different levels of depolarisation from the RMP. Data from (**a**) patch 2702st03.dat (sweeps 17–50, 83–116, 149–182 and 215–248), (**b**) patch 2702st10 (sweeps 17–50, 83–116, 149–182 and 215–248); *V*_0_ = −40, *V*_1_ = from +40 to +120, *V*_2_ = +100 mV relative to the resting potential for both data sets.

8-CPT did not produce major kinetic changes in the observed Na^+^ currents. This is illustrated by comparing time courses of the inward currents following the *V*_1_ voltage step before and following 8-CPT challenge. Figure 4a illustrates such currents, dimensioned in nA, obtained by signal averaging currents from the parts of the sweeps imposing *V*_1_ steps between (RMP + 40) and (RMP + 120) mV from a single patch employing the pulse protocol (a) before and (b) following challenge by 8-CPT. Figure 4c shows that traces normalised to their peak values obtained before (solid lines) and following (dotted lines) at different *V*_1_ excursions were superimposable at least in the initial stages following the depolarising steps. This was apart from small differences at deflections of 40 mV which are consistent with the shallower voltage dependence of both activation and inactivation observed in the presence of 8CPT.

### Control results from challenge with dantrolene in combination with 8-CPT

The above experiments thus demonstrated significant effects on Na^+^ current magnitudes following challenge by extracellular 8-CPT, in intact cardiomyocytes whilst minimising intracellular perturbations. In view of its lipophilic nature and its intracellular Epac-dependent site of action, reversibility of these actions was tested by challenge using dantrolene in combination with 8-CPT. Dantrolene is known to exert, similarly intracellular, actions directly blocking RyR-mediated release of SR Ca^2+^ whether added extracellularly or injected^36^. The final experiments that followed applied dantrolene challenge alone in the absence of 8-CPT, to control for any intrinsic actions of dantrolene on Na^+^ current. This strategy thus tested the reversibility of the intracellular effects of Epac activator on RyR mediated actions on Na^+^ channel function through matching this with applications of a known RyR antagonist. In addition, adopting this control strategy thus additionally directly confirmed a hypothesis attributing the observed 8-CPT actions on Na^+^ current to actions on the RyR-Ca^2+^ release channel.

**Figure 5:**
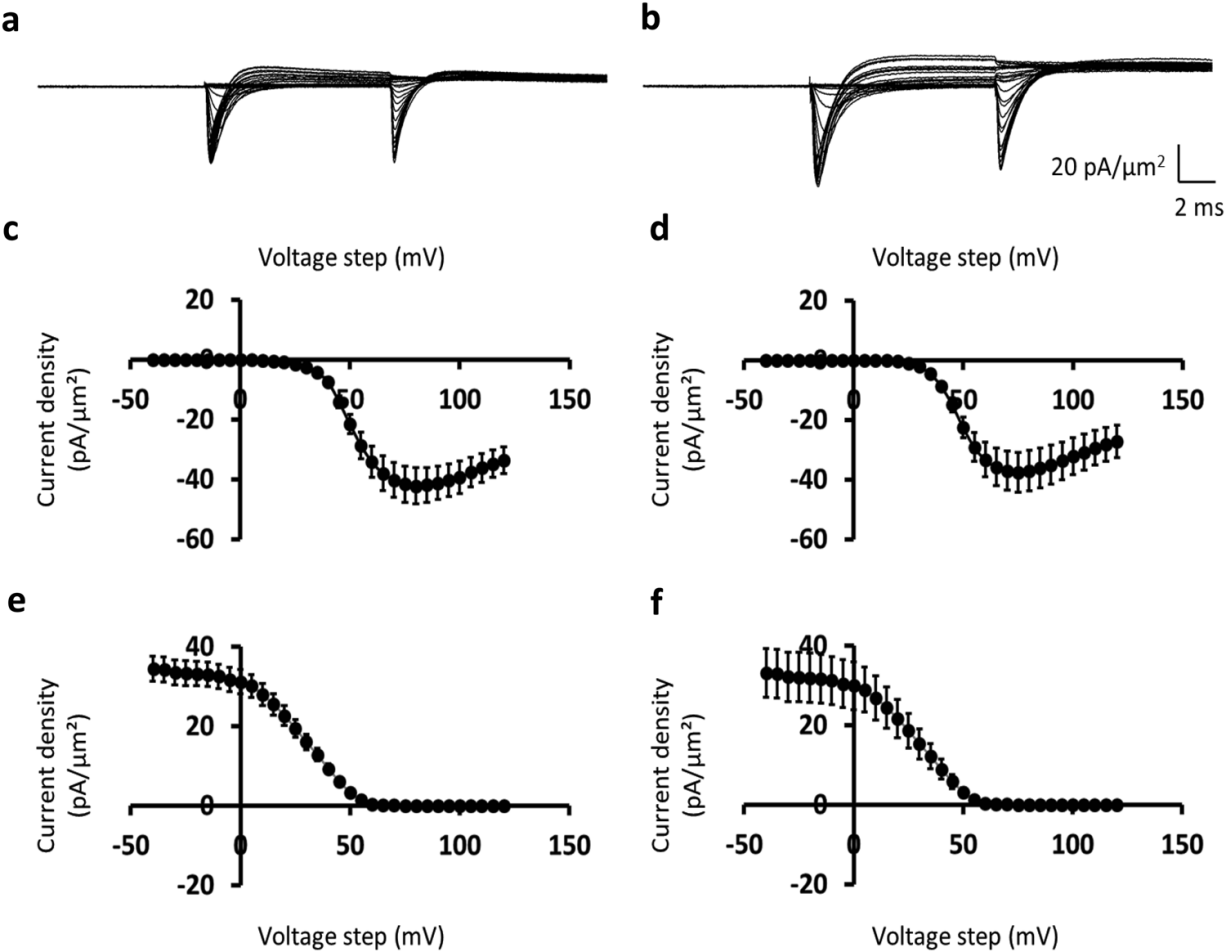
Inclusion of dantrolene abolishes the inhibitory effect of 8-CPT on inward currents. (**a**,**b**) Membrane ion currents (**a**) before and (**b**) following challenge by a combination of 8-CPT and dantrolene in response to pulse protocol consisting of *V*_1_ and *V*_2_ steps showing increased *I*_1_ and decreased *I*_2_ with increasingly depolarising *V*_1_ steps that were not affected by the pharmacological challenge. (**c**–**f**) Plots of peak inward currents, *I*_1_ and *I*_2_, in response to (**c**,**d**) *V*_1_ steps and (**e**,**f**) *V*_2_ steps respectively. These reflect the voltage dependences of inward current (**c**,**d**) activation and (**e**,**f**) inactivation (**c**,**e**) before and (**d**,**f**) following pharmacological challenge. Data from (**a**,**c**,**e**) patch 2102st03.dat (sweeps 1–66), (**b**,**d**,**f**) patch 2102st05 (sweeps 1–66); *V*_0_ = −40, *V*_1_ = from −40 to +120, *V*_2_ = +100 mV relative to the resting potential for both data sets.

The subsequent experiments accordingly explored the extent to which the effects outlined above could be abrogated by challenge with a combination of 8-CPT (1 *µ*M) and dantrolene (10 *µ*M). Dantrolene is a known RyR blocker. It could therefore be used to test for an involvement of RyR-mediated Ca^2+^ release in the effects of Epac activation on Na^+^ channel function, by exploring whether it would abrogate the effect of 8-CPT. In these control experiments, simultaneous administration of 8-CPT and dantrolene resulted in current amplitudes similar to those observed before pharmacological challenge, indicating that the effect of 8-CPT in inhibiting Na^+^ current was abolished (Fig. 5a,b). This was reflected in the activation and inactivation I-V curves shown in Fig. 5(c,d) and 5(e,f) respectively. Thus, fits of the appropriate Boltzmann expression to the activation data, before and following pharmacological challenge demonstrated similar values for *I*_max_ (−43.06 ± 6.07 pA/*μ*m^2^ (*n* = 8) vs. −37.60 ± 6.62 pA/*μ*m^2^ (*n* = 13); *t* = 0.54, φ = 19, *P* ≫ 0.05), V* (49.92 ± 1.44 mV (*n* = 8) vs. 46.79 ± 0.80 mV (*n* = 13); *t* = 1.96, φ = 19, *P* ≫ 0.05), and *k* (7.30 ± 0.62 mV (*n* = 8) vs. 6.32 ± 0.36 mV (*n* = 13); *t* = 1.39, φ = 19, *P* ≫ 0.05). Similar fits of Boltzmann functions to inactivation data gave concordant results for which *I*_max_ was 38.77 ± 5.47 pA/*μ*m^2^ (*n* = 8) and 32.62 ± 6.00 pA/*μ*m^2^ (*n* = 13) (*t* = 0.68, φ = 19, *P* ≫ 0.05); V* was 28.50 ± 3.19 mV (*n* = 8) and 25.57 ± 2.16 mV (*n* = 13) (*t* = 0.76, φ = 19, *P* ≫ 0.05); and *k* was 11.33 ± 0.49 mV (*n* = 8) and 11.31 ± 0.85 mV (*n* = 13) (*t* = 0.01, φ = 19, *P* ≫ 0.05). As treatment with dantrolene could reverse the effects of 8-CPT on Na^+^ current amplitudes, activation of RyR is likely involved in this 8-CPT-induced inhibition of Na^+^ currents.

### Control results from challenge with dantrolene alone

**Figure 6:**
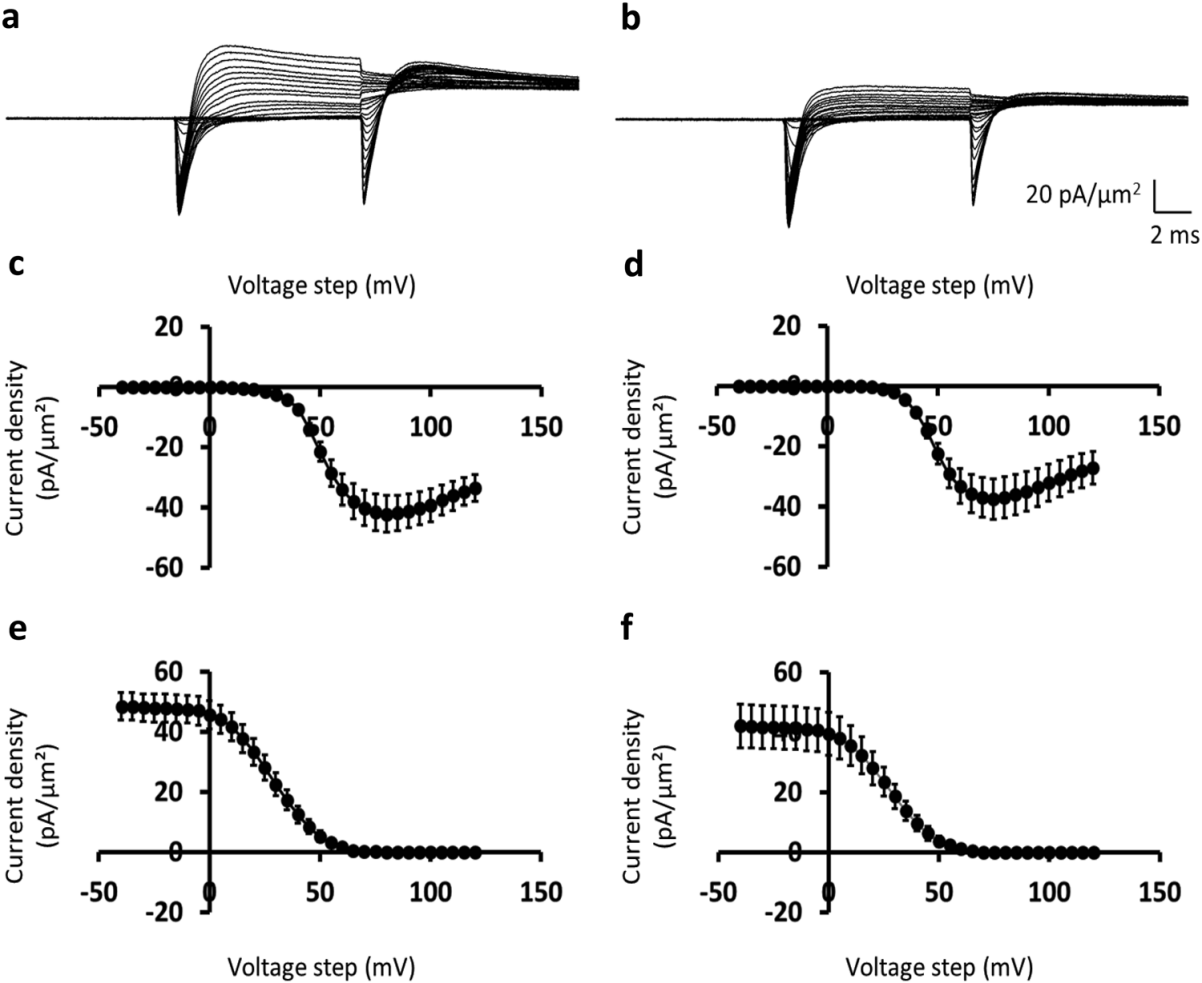
Inclusion of dantrolene alone leaves inward currents unchanged. (**a**,**b**) Membrane ion currents (**a**) before and (**b**) following challenge by dantrolene alone in response to pulse protocol consisting of *V*_1_ and *V*_2_ steps. Note increased *I*_1_ and decreased *I*_2_ with increasingly depolarising *V*_1_ steps that were not affected by the pharmacological challenge. (**c**–**f**) Plots of peak inward currents, *I*_1_ and *I*_2_, in response to (**c**,**d**) *V*_1_ steps and (**e**,**f**) *V*_2_ steps respectively. These reflect the voltage dependences of inward current (**c**,**d**) activation and (**e**,**f**) inactivation (**c**,**e**) before and (**d**,**f**) following pharmacological challenge. Data from (**a**,**c**,**e**) patch 2802st02.dat (sweeps 1–66), (**b**,**d**,**f**) patch 2802st06 (sweeps 1–66); *V*_0_ = −40, *V*_1_ = from −40 to +120, *V*_2_ = +100 mV relative to the resting potential for both data sets.

The above results are consistent with a hypothesis implicating a RyR-dependent Ca^2+^ release providing a mechanism by which Epac activation by 8-CPT compromised Na^+^ channel function. The final controls accordingly investigated the effect of RyR block by dantrolene (10 *µ*M) alone (Fig. 6). Analysis of the resulting experimental traces before (a) and following (b) dantrolene treatment (Fig. 6a,b) demonstrated that dantrolene did not exert any direct effects on Na^+^ current activation or inactivation. Thus, challenge by dantrolene alone produced results closely similar to findings following combined 8-CPT and dantrolene challenge. Analysis of activation curves (Fig. 6c,d) gave activation curve values of *I*_max_, V*, and *k* that were unchanged before and following dantrolene challenge. They gave for *I*_max_, −54.54 ± 4.44 pA/*μ*m^2^ (*n* = 7) and −53.60 ± 9.26 pA/*μ*m^2^ (*n* = 7) (*t* = 0.08, φ = 12, *P* ≫ 0.05); V*, 49.43 ± 1.53 mV (*n* = 7) and 45.91 ± 1.78 mV (*n* = 7) (*t* = 1.39, φ = 12, *P* ≫ 0.05); and *k*, 5.63 ± 0.54 mV (*n* = 7) and 5.53 ± 0.61 mV (*n* = 7) (*t* = 0.12, φ = 12, *P* ≫ 0.05). Similarly, the inactivation data (Fig. 6e,f) gave for *I*_max_, 49.42 ± 4.44 pA/*μ*m^2^ (*n* = 7) and 41.91 ± 7.24 pA/*μ*m^2^ (*n* = 7) (*t* = 0.99, φ = 12, *P* ≫ 0.05); V*, 30.40 ± 1.72 mV (*n* = 7) and 26.75 ± 2.20 mV (*n* = 7) (*t* = 1.46, φ = 12, *P* ≫ 0.05); and *k*, 10.19 ± 0.66 mV (*n* = 7) and 9.91 ± 0.53 mV (*n* = 7) (*t* = 0.37, φ = 12, *P* ≫ 0.05). Therefore, as dantrolene alone was unable to affect Na^+^ currents, the reversal of 8-CPT action by dantrolene is due to interference of the inhibitory action of 8-CPT and not by the direct actions of dantrolene on Na^+^ currents.

## Discussion

The present experiments demonstrate for the first time a downstream modulation of skeletal muscle Nav1.4 function by the recently characterised Epac signalling system, brought about by its action on RyR1-mediated release of intracellularly stored Ca^2+^. Intense recent interest has been directed at such PKA-independent activation mechanisms in distinct critical cell physiological processes. Yet there have been no studies of their actions on excitable as opposed to metabolic function^1,2^ in skeletal muscle, particularly its RyR1-mediated signalling, or further downstream effects on Nav1.4 function. Although such studies on Epac action have been made on cardiac myocytes^4–6^, skeletal muscle activation is distinct in its involving direct charge-coupled as opposed to Ca^2+^-induced Ca^2+^ release by RyR1 rather than RyR2, driven by surface membrane Cav1.1 as opposed to Cav1.2 activation following depolarisation, that does not involve Ca^2+^ current. Furthermore, these events are initially driven by Nav1.4 rather than Nav1.5 opening^19^. The present experiments demonstrated inhibitory effects on skeletal muscle Nav1.4 activation by the Epac activator 8-CPT. These were reversed by the RyR-blocker dantrolene. Yet dantrolene challenge by itself did not affect Na^+^ current. The observed effects on Nav1.4 kinetics, and activation and inactivation steady state properties were quantifiable in terms of maximum currents, steepness factors and half-maximal voltages.

The experimental procedures used loose patch clamp methods, apposing micropipettes on the surface membranes of intact skeletal myocytes. This formed relatively low resistance, <~2 MΩ, seals^37^ avoiding membrane or cellular disruption thereby leaving intracellular, particularly [Ca^2+^]_i_, homeostasis unperturbed. This approach can be applied to intact cells in tissue preparations *in situ* and further permits pipette re-use in successive recording protocols^38,39^. Currents from the same pipette before and after drug treatment could therefore be compared at multiple recording sites within the same *in situ* muscle fibre. In contrast, conventional tight seal patch clamping methods often include Ca^2+^-sequestrating ethylene glycol-bis(β-aminoethyl ether)-N,N,N’,N’-tetraacetic acid (EGTA) in the pipette solutions that could perturb intracellular Ca^2+^ homeostasis, often require isolated or cultured cells and enzyme-cleaning of membranes, and involve pipette replacement between successive patches.

The cAMP analogue and Epac agonist 8-CPT^3^ was applied at concentrations (~1 µM) considered 300-fold preferentially selective for Epac over PKA activated pathways^3,40–42^. Although 8-CPT also inhibits phosphodiesterase isoforms, it only does so at considerably higher concentrations^43^. Firstly, in common with the previous findings in murine cardiac, atrial and ventricular muscle^4^, 8-CPT reduced skeletal muscle cell Na^+^ current amplitudes whether quantified from peak currents, *I*_max_, or measurements from both activation and inactivation current-voltage curves. 8-CPT also increased their corresponding steepness factors, *k*, reflecting decreased Na^+^ channel voltage sensitivities. Secondly, superimposition of scaled membrane currents before and after 8-CPT challenge suggested that 8-CPT contrastingly did not affect Na^+^ current kinetics, other than modest changes corresponding to the alteration in Na^+^ channel voltage sensitivity.

These effects of 8-CPT were abrogated by further addition of dantrolene, in parallel with the previous study in cardiac muscle cells^4^. Dantrolene is a ryanodine receptor (RyR)-sarcoplasmic reticular (SR) Ca^2+^ channel blocker acting through stabilising RyR closed states by enhancing interactions between its N-terminal and the central domains particularly under conditions of increased open channel probability^44–46^. In cardiac muscle, dantrolene inhibited RyR2-mediated diastolic Ca^2+^ release, decreasing frequencies and durations of aberrant Ca^2+^ sparks in cardiomyocytes modeling catecholaminergic polymorphic ventricular tachycardia (CPVT)^46^ and cardiac failure models^45,47^. Its observed action thus implicated mechanisms involving RyR-mediated SR Ca^2+^ release in the observed 8-CPT action on Nav1.5. Furthermore, challenge with dantrolene alone did not affect either Na^+^ current amplitude or values of *k*. This excludes actions of dantrolene arising from any direct effects on Na^+^ current. Thus, both dantrolene and 8-CPT likely produced their respective effects upon skeletal muscle Nav1.4 through a common Ca^2+^-dependent mechanism. Through these pharmacological procedures, voltages of half-maximal activation, V*, for both activation and inactivation were unchanged. Thus, certain regulatory processes that act on cardiac Nav1.5 may well also do so in skeletal muscle Nav1.4.

The present findings extend signalling schemes involving Epac-dependent cAMP-triggered activation pathways suggested for cardiac muscle to a similar regulation of Na^+^ channel function in skeletal muscle. First, they suggest that skeletal muscle also possesses an Epac signalling pathway capable of influencing ion channel function. Epac is a guanine nucleotide exchange factor (GEF) for the Ras-like small GTPases Ras-related protein (Rap)1 and Rap2^48^. Of the three Epac1, Epac2 and Related to Epac (Repac) isoforms^3^, Epac2 has been associated with regulating Ca^2+^-dependent processes^1,2,48^, including pancreatic β-cell excitation-secretion coupling^49–53^ and activation of spontaneous transient outward currents in vascular smooth myocytes^54^. In cardiac muscle, pro-arrhythmic effects of Epac activators on both spontaneous SR Ca^2+^ release and diastolic Ca^2+^ transients^6^ were abolished by genetic ablation particularly of Epac2, as well as of β_1_ adrenoreceptor, Ca^2+^/calmodulin-dependent protein kinase II-δ (CaMKIIδ), and with RyR2-S2814 phosphorylation^12^. These effects may involve a novel pathway in which an activated Epac upregulates Rap1 action in turn stimulating phospholipase C (PLCε) mediated phosphatidylinositol 4,5-bisphosphate (PIP_2_) hydrolysis to diacylglycerol (DAG)^2,55^. DAG triggers a protein kinase C (PKC) mediated CaMKII activation in turn promoting a RyR2 phosphorylation associated with increased SR Ca^2+^ release. PLCε also exerts GEF activity that amplifies Rap1 activation initiating further, positive feedback, activation of PLCε. Conversely, CaMKII inhibition abolished such Epac-induced alterations in Ca^2+^ homeostasis^6^.

Secondly, the findings are consistent with the CaMKII activation phosphorylating skeletal muscle RyR1 as it does cardiac muscle RyR2. These effects are associated with increased SR Ca^2+^ release and cytosolic Ca^2+^. This is consistent with their conserved CaMKII phosphorylation sites at potentially regulatory locations in the RyR1/RyR2 loop connecting their third and fourth repetitive sequences^56^. Epac activation may also induce inositol 1,4,5-triphosphate (IP_3_) receptor (IP_3_R)-mediated SR Ca^2+^ release^57^, likely via IP_3_ generation from PIP_2_ by PLCε.

Thirdly, the present findings directly confirm previous *in vitro* suggestions that the resulting increased [Ca^2+^]_i_ may then inhibit Nav1.4 function in intact skeletal myocytes *in situ*^25,27^ in common with its action on cardiac Nav1.5^4,58^. Nav1.4 and Nav1.5 isoforms possess similar amino acid sequences consistent with such multiple structural and functional homologies^13^. In common with cardiac Nav1.5, Ca^2+^ could then regulate Nav1.4 through a number of possible mechanisms. Thus: (a) an EF-hand motif in the Nav1.5 C-terminus domain was reported to directly bind Ca^2+^ with an affinity typical of Ca^2+^-sensor proteins^14^. Such EF-hand-like domains also occur in Nav1.4. (b) Ca^2+^ could bind at EF-hand motifs of CaM that thereby acts as a Ca^2+^ sensor. The CaM could then bind to an isoleucine-glutamine (IQ) domain conserved between Nav1.4 and other Nav isoforms. Thus, CaM co-expression with Nav1.4 negatively shifted the steady-state voltage-dependences of Na^+^ current activation, an effect abolished both by expression of a mutant CaM with impaired Ca^2+^ binding^28^ and by mutation of the Nav1.4 IQ domain^29^. The mutant Nav1.4 remained Ca^2+^-sensitive, suggesting persistent additional Ca^2+^-mediated mechanisms regulating Nav1.4 gating independent of Nav1.4 IQ domains. Thus: (c) Nav1.4 and Nav1.5 share sites phosphorylated by CaMKII action^17^ to extents dependent on [Ca^2+^]_i_. CaMKII activation downstream of the Epac2/Rap1/PLCε cascade could also phosphorylate Nav1.4 independently of Ca^2+^ release. Additionally, CaMKII is itself regulated by Ca^2+^/CaM. (d) Nav1.4 can also be phosphorylated by PKC resulting in reduced Na^+^ current amplitudes *in vitro*^59^. Similar activation of PKC by the Epac2/Rap1/PLCε cascade may thus also directly contribute to the inhibition of Na^+^ currents. (e) Ca^2+^ may produce longer-term inhibition of Na^+^ channel expression: gain of function, *RyR2-P2328S*, mutations appear to downregulate cardiomyocyte Nav1.5 expression^60^.

Clinical evidence suggests that a [Ca^2+^]_i_-dependent inhibition of Nav1.4 could be physiologically important in regulating skeletal muscle excitability. Nav1.4 dysfunction has been reported following mutations in its encoding *SCN4A* gene. This may be implicated in skeletal muscle disorders including hyperkalaemic periodic paralysis, K^+^-aggravated myotonia^61^ and sudden infant death syndrome^24^. In particular, *SCN4A* abnormalities resulting in Ca^2+^-dependent Na^+^ channel dysregulation have been associated with cold- and K^+^-aggravated myotonias^25^. Thus, a Nav1.4 mutation localised to the EF-hand-like domain was observed in a patient with K^+^-aggravated myotonia^62^. Patch-clamp analysis of Na^+^ currents from such mutant channels have showed impaired inactivation and slowed Na^+^ current kinetics^62^. Small defects in Nav1.4 inactivation, which can also occur following weakening of Ca^2+^-dependent inhibition, can also predispose to myotonias^61^. Finally, the resulting inhibition of myocyte excitability may also contribute to weakness in dystrophic muscle, where resting [Ca^2+^]_i_ is constitutively substantially raised^63^.

## Materials and Methods

This research has been regulated under the Animals (Scientific Procedures) Act 1986 Amendment Regulations 2012 following ethical review and approval by the University of Cambridge Animal Welfare and Ethical Review Body (AWERB). Chemical reagents used were purchased from Sigma-Aldrich (Poole, UK) unless otherwise stated. Muscle preparations were obtained from C67BL6 wild-type mice housed in a licensed facility at room temperature, given free access to sterile rodent chow and water, and exposed to 12 hour light/dark cycles. Mice were killed by cervical dislocation immediately before use by Home Office-licensed personnel, according to Schedule 1 of the UK Animals (Scientific Procedures) Act (1986). Gastrocnemius and soleus muscles were isolated and dissected free of connective tissue. They were bathed in Krebs–Henseleit (KH) solution (mmol/L: NaCl, 130; KCl, 4.0; HEPES, 1.2; MgCl_2_, 1.0; CaCl_2_, 1.8; glucose, 10; and Na-pyruvate, 2.0; pH adjusted to 7.4) during dissection. Isolated muscles were transferred intact into the Sylgard-bottomed experimental bath and secured with A1 insect pins.

Experiments were performed in the following variants of the basic KH solution into which were introduced dimethyl sulfoxide (DMSO) vehicle containing 8-(4-chlorophenylthio)-2′-O-methyladenosine 3′,5′-cyclic monophosphate sodium salt (8-CPT) (BIOLOG Life Science Institute, Bremen, Germany) and/or dantrolene sodium (LKT Laboratories Inc, St Paul, MN, USA), or neither agent. Solutions thus contained: (1) KH control solution, (2) KH + 8-CPT (1 *μ*mol/L) (3) KH + 8-CPT (1 *μ*mol/L) + dantrolene (10 *μ*mol/L) and (4) KH + dantrolene (10 *μ*mol/L), with <0.02% DMSO vehicle in all cases. These solutions were first filtered to remove particles with a diameter greater than 10 *μ*m using standard filtration paper (Millipore, Bedford, MA, USA). Control of the bath temperature was important as Na^+^ conductance is dependent on temperature^64^. Bath temperature was controlled at 24–26 °C by circulating heated water through a coil in the bath, a temperature range chosen to optimise Na^+^ currents observed as well as preparation lifespan. The bath solution was replaced with fresh KH solution every 30 min to prevent metabolite accumulation around the muscle preparation and obviate evaporation.

A Flaming/Brown micropipette puller (model P-97, Sutter Instrument Co. Novato, CA, USA) was used to pull pipettes from borosilicate glass capillaries (catalog no GC150-10: Harvard Apparatus, Cambourne, Cambs, UK) for the loose patch clamp studies. The pipettes were visualised under a microscope at 250x magnification and the tips scored using a diamond knife to form a small groove. A force was then applied distal to the groove. This caused the pipette tip to break off perpendicular to the long axis of the pipette. The squarely-broken tips were visualised at 400x magnification and fire-polished with an electrically-heated nichrome filament to smooth the edges of the tip. The internal diameter of the pipettes was measured at 400x magnification. Only pipettes with tips which had a very smooth edge perpendicular to the long axis of the pipette and internal diameters of 25–30 *µ*m after polishing were selected. The pipette was then bent about 1 mm from its tip to an angle of ~45° relative to its long axis, to allow the pipette tip to contact the membrane of the muscle preparation at 90° when mounted on the recording amplifier head stage, as depicted in Fig. 1a. The pipettes were mounted on to the pipette holder in the experimental setup. The distal half of the micropipette was filled with the KH solution from the bath, with the help of suction provided by a syringe via an air-filled connection with the pipette holder. The bath was actively grounded at reference potential to complete the circuit. Ag/AgCl electrodes were used to provide a reversible electrical connection between the bath solution and the physical electronic circuit.

For loose patch clamp studies, the pipette tip was lowered perpendicular to the membrane of the muscle preparation. A gentle suction was applied to form a seal around the patch of membrane under the pipette. The technique thus does not involve impalement of the cell membrane in an accordingly intact muscle fibre (Fig. 1b). The current flowing across this patch of membrane drawn into the pipette tip could thus be measured by the recording electrode in the pipette, relative to the actively-grounded reference potential of the bath. The potential across the membrane within the patch then corresponds to the cell resting membrane potential (RMP) prior to application of the pulse protocols. The pulse protocols then clamped the voltage of the fluid within the pipette through their sequence of command potentials. This in turn accomplished the required changes in potentials across the membrane within the patch. As the voltages are thus applied from the extracellular rather than the intracellular space, a negative voltage step represents hyperpolarisation and a positive voltage step represents depolarisation of the patch relative to RMP. Membrane potentials in this paper are thus described relative to the RMP, and imposed voltage changes described as changes in intrapipette potential.

The equivalent circuit of a typical patch is shown in Fig. 1b. The relatively low seal resistance, of typically <2 MΩ in this loose as opposed to conventional gigaseal patch clamp technique, resulted in a substantial leak current. This ohmic leakage, together with the current flowing through the pipette capacitance, was largely subtracted by the custom-built electronic circuitry of the loose patch clamp amplifier, which also corrected for the effects on the clamped membrane potential of pipette series resistance. The remaining leakage currents were then corrected for using a P/4 leak protocol. During this protocol which was applied following the test steps themselves, four voltage steps of a quarter of the amplitude and of opposite sign were applied to the membrane patch. These were of a sign and/or amplitude which would not activate the voltage-gated conductances, thus representing the leak currents only. The responses were measured, summed and subtracted from the currents obtained from the larger original voltage steps, thus correcting for any residual linear leakage currents not already removed by the voltage clamp circuit.

An IBM-compatible computer was used to deliver voltage clamp steps relative to the RMP. To detect the presence of ion channels in the membrane patch being studied, depolarising pulses of (RMP + 100) mV lasting 15 ms were first applied. Only patches which produced clearly resolved inward currents with kinetics characteristic of Nav channels were selected. A double pulse protocol (Fig. 1c) was then used to assess Na^+^ current activation and inactivation properties in a single sweep. The voltage-dependent activation and inactivation properties of Na^+^ currents could thus be investigated before and following introduction of the various drugs. More detail on the pulse protocol is discussed in the Results section. Data was sampled at a 50 kHz digital sampling rate and filtered with a DC-10 kHz bandwidth, using a 10 kHz Bessel low pass filter. The currents obtained from the double pulse protocol were normalised to the pipette tip cross-sectional area to give current densities, using the formula:

$${\rm{current}}\,{\rm{density}}\,(\mathrm{pA}/{\mu }{{\rm{m}}}^{2})=\frac{{\rm{current}}\,{\rm{measured}}\,(\mathrm{nA})\times {\rm{1000}}}{{\rm{\pi }}\times {[\mathrm{pipette}{\rm{radius}}({\mu }{\rm{m}})]}^{2}}$$

Analysis of each pharmacological condition compared data collected during the treatment with the drug(s) with data collected before treatment, employing Student’s unpaired 2-tailed *t*-test to a significance level of *p*-value (*P*) < 0.05. Pre-treatment data was kept in three separate groups depending on the subsequent pharmacological treatment due to the large variation in Na^+^ currents observed between muscle preparations. As the same muscle preparations were used to collect data before and during treatment with the drug(s), comparisons before and during drug treatment could be made. Curve fitting procedures applied to activation and inactivation current voltage curves were carried out by the open source fitting algorithms QtiPlot (Version 0.9.8.9 svn 2288).

### Ethical approval

This research has been regulated under the Animals (Scientific Procedures) Act 1986 Amendment Regulations 2012 following ethical review and approval by the University of Cambridge Animal Welfare and Ethical Review Body (AWERB). All procedures were completed by Home Office-licensed personnel and fell within the scope of Schedule 1 of the UK Animals (Scientific Procedures) Act (1986).

## Data Availability

The datasets generated during and/or analysed during the current study are available from the corresponding author on reasonable request.
